# Economic burden of drought using the life satisfaction approach: A case study of slum dwellers in southeast Iran

**DOI:** 10.1371/journal.pone.0340300

**Published:** 2026-01-06

**Authors:** Minoo Mohammadkhani, Nouzar Nakhaee, Reza Goudarzi, Mahmood Nekoei-Moghadam

**Affiliations:** 1 Department of Health Education and Health Promotion, School of Health, Occupational Environment Research Center, Rafsanjan University of Medical Sciences, Rafsanjan, Iran; 2 Health Services Management Research Center, Institute for Futures Studies in Health, Kerman University of Medical Sciences, Kerman, Iran; 3 Health Services Management Research Center, Institute for Futures Studies in Health, Kerman University of Medical Sciences, Kerman, Iran; 4 Health in Disasters and Emergencies Research Center, Institute for Futures Studies in Health, Kerman University of Medical Sciences, Kerman, Iran; Nuclear Science and Technology Research Institute, IRAN, ISLAMIC REPUBLIC OF

## Abstract

Drought is one of the most visible effects of climate change and poses significant challenges for sustainable development. Assessing the costs of drought is essential for effective policymaking, and indirect costs are likely to provide a more comprehensive estimate. This descriptive-analytical study was conducted in 2023 among households in the slum areas of Kerman, a city in southeastern Iran. Given the population’s diversity, a cluster sampling method was employed, and data were collected from 507 households through a questionnaire. The research team estimated the Ordinary Least Squares (OLS) model, the Marginal Rate of Substitution (MRS), and Willingness to Pay (WTP) using the life satisfaction approach. The results indicated that 38.42% of households identified drought as the primary reason for their migration, while 33.27% were formerly smallholder farmers or agricultural workers. Notably, farmers and ranchers reported significantly lower life satisfaction levels. The MRS for these households was −0.570, underscoring the negative impact of drought on their income. Furthermore, based on their income, their willingness to pay for drought mitigation was estimated at $1,968.98 per household, reflecting their economic constraints. Overall, the modeling results from the life satisfaction approach indicate that drought imposes a significant economic burden on households, particularly smallholders and agricultural workers. The forced migration to the slum areas of Kerman, driven by declining agricultural potential viability, has not produced stability but has instead led to ongoing economic instability and reduced life satisfaction.

## 1. Introduction

Climate change poses significant challenges to sustainable development, impacting social, economic, political, cultural, and environmental dimensions. It is seen as an obstacle to socio-economic growth and a contributing factor to poverty, particularly in underdeveloped and developing countries [1–3]. One of the most notable effects of climate change is drought, which is expected to become more frequent and severe, especially in semi-arid regions experiencing water stress [4,5]. According to a World Bank report, as many as 216 million people could become internal climate migrants by 2050 (under the pessimistic scenario), largely due to drought, with economic, environmental, and social repercussions, and threatening development gains [6,7].

Direct economic damages caused by drought are tangible and relatively straightforward to measure, but indirect and intangible damages are more challenging to quantify and are often underestimated [8,9]. For instance, a severe drought in California in 2006 caused $4.4 billion in damage. In contrast, the drought in the Horn of Africa from 2010 to 2011 led to 250,000 deaths and left millions reliant on humanitarian aid [10]. Drought in Iran has also posed significant socio-economic challenges for households. Between 2006 and 2008, damages amounting to 19 billion USD were reported in the agricultural sector alone. Therefore, drought affects income, savings, and employment opportunities, particularly among vulnerable households such as farming rural families. As a result, many households are often compelled to migrate to urban areas in search of better living conditions. However, migration to cities and the consequent increase in urbanization, in the absence of adequate infrastructure, frequently lead to the expansion of informal settlements or slums on the outskirts of urban areas [11–14].

Slum-dwelling households experience vulnerability differently from rural or national populations, as they often lack formal land ownership. This absence of legal tenure deprives them of access to governmental support and limits their opportunities for stable income, water supply, and other essential services compared with other areas. Consequently, due to inadequate infrastructure, insufficient essential services, and the lack of proper adaptation measures, climate-related risks such as drought are intensified among residents of these settlements [15]. Moreover, certain socio-economic factors, including large household sizes and long distances from provincial centers, have made the southeastern provinces—particularly Kerman Province—more vulnerable to drought than other regions [16].

Environment quality is an important political issue with a significant impact on individual well-being. Therefore, understanding the impacts of drought and assessing the associated costs is crucial for effective policymaking. While estimating indirect damages can provide a more comprehensive perspective, the challenges, expenses, and time-consuming nature of non-market valuation methods often lead to these damages being overlooked [7,8,17]. However, new approaches, such as the Life Satisfaction (LS) Approach, have introduced a fresh perspective in subjective well-being research within economics, allowing researchers to quantify the monetary value of non-market factors. Subjective well-being can serve as a practical indicator of individual welfare. This approach enables a deeper understanding of how drought impacts individual well-being beyond traditional economic measures [9,18,19].

Life satisfaction is a mental construct that individuals create based on their criteria and past experiences. The LS approach has certain advantages over traditional methods. Firstly, it relies on self-reported evaluations rather than observed behaviors, which imposes fewer restrictive assumptions and allows previously difficult qualities to be quantified. Secondly, respondents are not directly asked to assign monetary values to public goods. Instead, they evaluate their overall life satisfaction. This approach reduces response biases and minimizes the potential for strategic behavior, as there is no incentive for respondents to give answers that might favor the questioner. The LS approach also considers monetary and non-monetary psychological costs [9,19].

Among the environmental issues examined using this approach are air pollution, water pollution, climatic conditions, and flooding [20]. Based on a literature review across various databases, we found only one study—Carroll’s Australian study—that applied the life satisfaction approach to the valuation of drought. This study highlights that the psychological costs of drought, arising from increased uncertainty and mental stress, are highly significant and nearly equivalent to its direct economic costs. Traditional methods, however, are likely to underestimate the total costs of droughts by neglecting these non-market and psychological impacts. One limitation of Carroll’s study was the insufficient data to distinguish between farmers and non-farmers [21].

Given the projected intensification of droughts, estimating the indirect and often overlooked damage they cause—particularly at the micro level—is essential. Our study contributes to the global literature by extending the Life Satisfaction Approach (LSA) to measure the economic burden of drought among vulnerable populations in southeastern Iran. Focusing on Kerman Province, whose economy is heavily dependent on agriculture and has been affected by frequent and prolonged droughts [22], we assume that drought negatively affects life satisfaction and income, especially among urban slum households due to their higher vulnerability. Accordingly, we address the following research questions and provide new insights into how climate change interacts with urban marginalization in developing regions:

What is the estimated magnitude of the drought’s impact on the life satisfaction of households in slum areas?
What is the estimated magnitude of the drought’s impact on the income of households in slum areas?

## 2. Materials and methods

### 2.1 Study design

This descriptive-analytical study was conducted in 2023 to examine the impacts of drought on household income and life satisfaction in Kerman, a city in southeastern Iran. Life satisfaction (LS) was the primary outcome variable, and its association with drought and other socioeconomic factors was analyzed.

### 2.2 Setting and participants

The study was conducted in the informal settlements of Kerman, a city in southeastern Iran, where drought is a key factor contributing to marginalization. Kerman has experienced an uncontrolled influx of population and migration due to rapid economic changes and prolonged drought conditions. This situation has resulted in unmanaged physical expansion of the city and the emergence of slum areas. Generally, residents of these neighborhoods are the first to experience water shortages and often lack essential urban infrastructure, making them more vulnerable to drought impacts [23].

Kerman’s slum areas consist of three districts: Chardah Masoom (District 1), the Sanaty (District 2), and the Seyedi (District 3), which together have a combined population of approximately 35,100 people. Specifically, the populations are as follows: 11,499 residents in the Sanaty District, 12,363 in the Seyedi District, and 11,238 in the Chardeh Masoom District [24]. The cluster sampling method was employed to ensure representativeness and reduce selection bias across Kerman’s three slum districts. Initially, marginal neighborhoods were identified, followed by selecting blocks within each neighborhood. Clusters within these blocks were determined, and households were randomly selected for the study.

### 2.3 Questionnaire/ Measured variables

The dataset for this study includes socio-economic factors that influence life satisfaction, indicators of water stress in households’ current residential areas, and meteorological data. A comprehensive questionnaire developed by researchers was used to assess various social, economic, and demographic factors, as well as health, religiosity, drought perception, and water stress, all of which impact life satisfaction in the districts. The questionnaire items were designed based on a thorough review of relevant literature on life satisfaction, existing questionnaires on social capital and status [25,26], health [27], religiosity [28], and findings from a qualitative study conducted with households living in slums in the city during the summer of 2022 [13].

The primary dependent variable in this study is life satisfaction (LS), which was measured using a single-item scale ranging from 0, (indicating “very dissatisfied’)’ to 10 (indicating ‘completely satisfied’).’ Participants were asked, “In general, how do you feel about your life right now? Please rate your life satisfaction on a scale from 0 (very dissatisfied) to 10 (completely satisfied).” Social status was evaluated through dimensions—social relationships, solidarity, social support, social trust, security, and access to services — using 15 items. Three items concerning social relations, solidarity, and social trust were evaluated at three social levels (family, friends, and neighbors), with responses rated on a five-point Likert scale [25]. Economic status was assessed using 36 items related to household income, expenditures, and other financial indicators [29]. Water insecurity was measured using 21 items that capture households’ perceived experiences of inadequate, unreliable, or unsafe water access for daily domestic, social, and health-related needs [30]. The items corresponding to economic status, social capital and status, and water stress were aggregated using Principal Component Analysis (PCA) to construct composite indices for each dimension.

Religiosity was measured using a visual analog scale, with the question: “Overall, how religious do you consider yourself? Please rate your religiosity on a scale from 0 (not religious at all) to 10 (very religious). Health status was assessed using the EQ-VAS from the EQ-5D questionnaire, in which participants rated their current health on a scale from 0 (worst) to 100 (best).

#### 2.3.1 Validity and reliability.

The questionnaire’s validity was established through a review by university experts and research team members. Reliability was assessed using the test-retest method with a sample of 30 individuals over 14 days, resulting in an Intraclass Correlation Coefficient (ICC) of 0.95, indicating high reliability.

It is important to note that the LS index is recognized by international organizations like the WHO and the UN for health-related economic evaluations [31]. The validity and reliability of this questionnaire have been confirmed through international studies involving diverse populations [32], as well as through specific studies conducted in Iran [33].

### 2.4 Data collection

Data were collected randomly through house-to-house visits from 1 July to 31 August 2023. Meteorological data, including temperature and precipitation measurements, were sourced from the synoptic stations of the Meteorological Organization of Kerman Province, covering 30 years from 1991 to 2022.

Data was collected by interviewers who were employees of healthcare centers in the slum areas. These interviewers had experience in conducting interviews, were willing to collaborate, and were thoroughly familiar with the district. To prepare the interviewers, introductory sessions were held to familiarize them with the questionnaire’s scope and emphasize the importance of accuracy in data collection. To ensure the process and data collection were executed correctly, the researcher carried out random supervision and reviews.

### 2.5 Sample size

The study focused on households that had lived in the slum areas of Kerman city for at least 1 year but no more than 10 years at the time of the research. It included native households from Kerman city or county, as well as those who had migrated from other counties within the province. Households from other provinces and those with non-Iranian nationality were excluded from the study. Since the research concentrated on households, the sample size formula was based on the number of households (n_h_) [34]:


$$n_{h}=\left(z^{\text{2}}\right)(r)(\text{1}-r)\;(f)(k)/\;\;(p)\;\left(\tilde{n}\right)(e^{\text{2}})$$

- z: The statistic that indicates the desired level of confidence = 2

- r: Estimation of the main index being measured (based on the statistics of the year 2019, approximately 0.7 percent of the population has been affected by the drought in Kerman [35], but to have a sample size with the largest number, r equal to 0.5 was considered.)

- f: Sample design effect = 1.5

- k: Expected non-response rate = 0.14 (Based on the pilot sample)

- p: The proportion of the target population to the total population based on r (the main index) (approximately 0.045 based on the slum dwellers to the population of Kerman [36])

- n: Average household size (Based on a pilot study with a sample size of 30 households) = 4

- e: margin of error = 0. 1 r = 0.05

Ultimately, a sample of 525 households was obtained; due to non-response to some main questions, 18 households were excluded, and in the end, information from 507 households was collected.

### 2.6 Statistical analysis

#### 2.6.1 Modeling framework.

In this study, we used empirical modeling at the micro-level, employing life satisfaction (LS) as an indicator of subjective well-being (SWB), as recommended by Welsch, Kühling, and Frey et al. [9,20]. The primary analytical framework used ordinary least squares (OLS) regression to estimate the determinants of LS, which was selected for its interpretability and extensive use in the subjective well-being literature. Although subjective well-being data can exhibit non-normality and heteroskedasticity, OLS remains an appropriate baseline estimator when complemented by robust standard errors and diagnostic validation, as performed in this study. The model considers variations and distinctions across individual, temporal, and spatial dimensions. It is assumed that the LS of individual (i) is a linear function of several factors, including household economic status (E_i_), social status (S_i_), health (H_i_), religiosity (R_i_), drought (D_i_), and other individual characteristics (X_i_). The effects of these variables on LS were estimated using the following OLS regression specification:


$$LS_{i}=\alpha +\beta_{\text{1}}E_{i}+\beta_{\text{2}}D_{i}+\beta_{\text{3}}S_{i}+\beta_{\text{4}}H_{i}+\beta_{\text{5}}R_{i}+\beta_{\text{6}}X_{i}+\varepsilon_{i}$$

In this model, the estimated coefficient for drought offers a direct evaluation of its impact on mental well-being. Ultimately, using the model’s coefficients, it is possible to estimate the trade-off between income and drought, or the implicit willingness to pay (WTP). In other words, the income increase necessary to compensate for a specific reduction in well-being due to drought for a household can be calculated [9,19]. This method can be an alternative to traditional non-market valuation techniques in cost-benefit analysis [37]. In this study, these WTP estimates represent the “indirect costs” of drought—namely, the welfare losses that are not directly observed in market transactions but are reflected in reductions in individuals’ life satisfaction.

The marginal rate of substitution (MRS) between income and drought is defined as:


$$MRS=\frac{\partial LS/\partial D}{\partial LS/\partial E}=\frac{\beta_{\text{2}}}{\beta_{\text{1}}}$$

To determine a household’s WTP to reduce drought, the final marginal rate of substitution is multiplied by the income level.

We conducted a series of econometric models to assess the impact of drought on household life satisfaction. Initially, we examined the primary determinants of life satisfaction, excluding drought. Subsequently, the influence of drought was incorporated into the analysis through four distinct models, each employing a different operationalization of drought’s impact on households.

Model 1 focused on households’ perceptions of drought, comparing their current and past experiences. Model 2 categorized households based on the occupation of the household head, distinguishing between families affected by drought and those unaffected. Specifically, families were classified as drought-affected if the household head had previously worked in agriculture or livestock farming and was forced to migrate due to drought-induced declines in agricultural and livestock production. In Models 3 and 4, we adopted quantitative measures to capture drought conditions. Model 3 utilized deviations in rainfall at meteorological stations from the average rainfall over the past 30 years. Model 4 employed the Standardized Precipitation-Evapotranspiration Index (SPEI), on a 12-month scale, using data from synoptic meteorological stations to assess drought severity.

We employed both objective and subjective drought measures based on risk perception theory [38]. Objective indices (rainfall deviation, SPEI) capture the physical severity of drought and enable spatial comparability. Subjective measures (direct experience, risk perception) capture households’ lived experience shaped by vulnerability and coping capacity, which often predict well-being outcomes more strongly than objective metrics [39]. This dual approach allows us to test: (1) direct material impacts of physical drought severity, and (2) psychological and social pathways mediated by subjective experience. The integration of both measurement types provides a comprehensive understanding of the impacts of drought on life satisfaction.

#### 2.6.2 Endogeneity considerations.

To address potential endogeneity between income and life satisfaction—given the possible bidirectional relationship—Lewbel’s (2012) heteroskedasticity-based instrumental variable approach was applied [40]. This method generates internal instruments from model residuals under the presence of heteroskedasticity, enabling identification without external instruments. The overidentification (Hansen J) and underidentification (Kleibergen–Paap) tests were applied to verify the validity and strength of the generated instruments. While the model passed the overidentification test (p = 0.38), the Kleibergen–Paap F-statistic (F = 3.18) indicated potential weak instrument bias; thus, the results were interpreted cautiously and compared with the OLS baseline for robustness.

#### 2.6.3 Diagnostic tests.

We conducted diagnostic tests to assess OLS assumptions. While multicollinearity was not a concern (all VIF < 2.5), tests of residual normality (Shapiro–Wilk, Skewness/Kurtosis) indicated deviations from strict normality. This outcome is not unexpected; OLS regression is generally robust to moderate violations of the normality assumption, particularly in samples of our size (n = 507), where the central limit theorem ensures the asymptotic normality of estimators [41]. To further safeguard against misspecification, we employed heteroskedasticity-robust standard errors. Therefore, we interpret results cautiously but with confidence in their reliability.

### 2.7 Software and model comparison

The household survey data and meteorological variables were initially entered into Excel software. Subsequently, Stata 17 was used to analyze the effect of these variables on life satisfaction. Additionally, the SPEI index was calculated in RStudio. To select the optimal econometric model, we compared the model fits using R-squared, the F-test p-value, and the Akaike Information Criterion (AIC) and Bayesian Information Criterion (BIC).

### 2.8 Ethics statement

This study received ethical approval from the Ethics Committee of Kerman University of Medical Sciences [Ethics Code: IR.KMU.REC.1401.264]. Informed written consent was obtained from either the head of the household or their spouse prior to participation. For participants unable to read or write, the consent process was explained verbally, and a fingerprint or signature confirmed consent. All participants were assured of the confidentiality of their information, voluntary participation, and their right to withdraw from the study at any time without consequence. No minors were included.

## 3. Results

In most studies on life satisfaction, the OLS (Ordinary Least Squares) model has been utilized [9,42]. After reviewing the data and examining the assumptions, we opted for the OLS model as the best fit. This model found a significant and positive relationship between income and LS. Subsequently, using the income and drought coefficients from the OLS model, we calculated the trade-off (monetary value of drought) between drought and income while holding LS constant.

Table 1 presents the characteristics of the determinants of life satisfaction in three study areas and for the entire sample under investigation. As shown, the average self-reported life satisfaction score in our study sample (mean age of 39.49 ± 10.02, approximate household size 4) is 6.41 ± 2.31, and we observe almost the same level of life satisfaction across the three regions. The average scores for religiosity and health in the study population were 7.29 ± 2.26 and 54.29 ± 23.13, respectively.

**Table 1:** Determinants of life satisfaction in three study areas and in the entire sample under investigation.

Variable	District			Total (n=507)
	Sanati (n=139)	Seyedy (n=211)	ChahrdahMasoom (n=157)	
Life Satisfaction, mean±SD	6.73 ± 1.23	6.80 ± 2.23	5.59 ± 2.87	6.41 ± 2.31
Health, mean±SD	54.03 ± 14.29	56.22 ± 21.21	51.92 ± 30.63	54.29 ± 23.13
Religion, mean±SD	6.73 ± 1.68	7.46 ± 2.23	7.56 ± 2.64	7.29 ± 2.26
Age, mean±SD	39.71 ± 11.49	38.28 ± 10.21	40.90 ± 8.06	39.49 ± 10.02
Household Size, mean±SD	3.16 ± 1.33	3.75 ± 1.18	4.0 ± 1.18	3.67 ± 1.26
Annual Income, mean±SD	3849.32 ± 1148.50	3041.62 ± 1190.55	3648.72 ± 1008.41	3451.06 ± 1177.75
Gender, n (%)				
Male	50 (35.97)	84 (39.81)	64 (40.76)	198 (39.05)
Female	89 (64.03)	127 (60.19)	93 (59.24)	309 (60.95)
Education level, n (%)				
Lower than diploma	102 (73.38)	186 (88.15)	116 (73.89)	404 (79.68)
University Degree	37 (26.62)	25 (11.85)	41 (26.11)	103 (20.32)
Marital Status, n (%)				
Married	105 (75.54)	194 (91.94)	145 (92.36)	444 (87.57)
Divorced	12 (8.63)	5 (2.37)	2 (1.27)	19 (3.75)
Widow	17 (12.23)	8 (3.79)	6 (3.82)	31 (6.11)
Single	5 (3.60)	4 (1.90)	4 (2.55)	13 (2.56)
Migration Cause, n (%)				
Drought	75 (54.74)	72 (34.12)	47 (29.94)	194 (38.42)
Other	62 (45.26)	139 (65.88)	110 (70.06)	311 (61.58)
Occupation in the past, n (%)				
Smallholder farmer or agricultural worker	86 (61.87)	43 (20.48)	39 (25.00)	168 (33.27)
Non-farmer	53 (38.13)	167 (79.52)	117 (75.00)	337 (66.73)
Insurance status, n (%)				
No	1 (0.72)	11 (5.21)	9 (5.73)	21 (4.14)
Yes	138 (99.28)	200 (94.79)	148 (94.27)	486 (95.86)
Perceived severity of drought in the past, n (%)				
Never	25 (17.99)	113 (53.55)	74 (47.13)	212 (41.81)
Little	52 (37.41)	34 (16.11)	12 (7.64)	98 (19.33)
Moderate	50 (35.97)	14 (6.64)	28 (17.83)	92 (18.15)
Sever	9 (6.47)	13 (6.16)	21 (13.38)	43 (8.48)
Extreme	3 (2.16)	37 (17.54)	22 (14.01)	62 (12.23)
Perceived severity of drought in the slum, n (%)				
Never	2 (1.44)	0	3 (1.91)	5 (0.99)
Little	85 (61.15)	0	6 (3.82)	91 (17.95)
Moderate	42 (30.22)	5 (2.37)	35 (22.29)	82 (16.17)
Sever	3 (2.16)	47 (22.27)	92 (58.60)	142 (28.01)
Extreme	7 (5.04)	159 (75.36)	21 (13.38)	187 (36.88)

Note: Totals may vary across columns due to missing data.

As indicated in Table 2, an increase in age and individuals whose spouse has passed away were statistically significant variables in the reduction of life satisfaction (β_age_ = −0.024, *p* = 0.020, and β_widow_ = −0.897, *p* = 0.031, respectively). In contrast, variables such as health (β = 0.021), religiosity (β = 0.201), social status (β = 0.444, *p* = 0.016), and economic status (β = 0.936, *p* = 0.001) showed a positive and significant correlation with life satisfaction. In the analysis excluding the economic factor, water stress in the current residence significantly decreased by 0.037 in life satisfaction (*p* = 0.024). However, after accounting for all factors in the multivariate analysis, this decrease was found to be insignificant.

**Table 2:** Results of OLS models without drought effects and with various definitions of drought.

OLS												
Variable	Primary Model		Final Model without Drought Effect		Model with Drought Perception		Model with drought effect based on Job of head		Model with rainfall changes		Model based on SPEI classification	
	Coef	SE	Coef	SE	Coef	SE	Coef	SE	Coef	SE	Coef	SE
Gender												
Female	−0.147	0.208	-	-	-	-	-	-				
Age	−0.028^*^	0.011	−0.024^*^	0.010	−0.023^*^	0.010	−0.024^*^	0.010	−0.024^*^	0.011	−0.018	0.013
Marital Statue Ref: Singel												
Married	0.165	0.605	-	-	-	-	-	-	0.740^*^	0.361	0.821^*^	0.367
Divorced	−0.689	0.785	−0.791	0.542	−0.807	0.545	−0.860	0.545	-	-	-	-
Widow	−0.745	0.729	−0.897^*^	0.415	−0.964^*^	0.425	−1.071^*^	0.421	-	-	-	-
Education												
University	−0.060	0.280	-	-	-	-	-	-	-	-	-	-
Living Place Ref: Sanati												
Seyedi	0.220	0.355	-	-	-	-	-	-				
Chahrdahmasoom	−0.906^**^	0.324	−1.066^***^	0.220	−0.967^***^	0.222	−1.110^***^	0.220				
Staytime	0.004	0.036	-	-	-	-	-	-				
Religion	0.217^***^	0.045	0.201^***^	0.043	0.212^***^	0.044	0.217^***^	0.044	0.180^***^	0.049	0.144^**^	0.052
EQ-VAS	0.022^***^	0.005	0.021^***^	0.005	0.023^***^	0.005	0.022^***^	0.005	0.024^***^	0.006	0.026^***^	0.006
Economic Status	0.863^**^	0.300	0.936^**^	0.277	0.780^**^	0.284	0.808^**^	0.284	1.004^**^	0.321	0.922^**^	0.334
Scocial Status	0.363	0.203	0.444^*^	0.183	0.463^*^	0.194	0.387^*^	0.190	0.671^**^	0.211	0.595^**^	0.220
Water Shortage	−0.031	0.025	-	-	−0.045^*^	−0.019	−0.021	0.016				
Drought	-	-	-	-	−0.166^*^	0.068	−0.461^*^	0.222	−0.004^*^	0.002	−0.903^*^	0.369
Constant	4.983^***^	0.839	5.175^***^	0.586	4.673^***^	0.612	5.407^***^	0.600	−1.313	2.513	4.025^***^	0.777
Goodness of fit												
R^2^	0.2361		0.2317		0.2466		0.2416		0.2091		0.1928	
F (p-value)	9.58 (0.0001)		16.59 (0.0001)		14.31 (0.0001)		13.96 (0.0001)		13.26 (0.0001)		10.99 (0.0001)	
AIC	1913.12		1903.692		1895.663		1901.863		1557.098		1435.813	
BIC	1974.726		1940.655		1940.815		1947.04		1588.165		1466.206	

*P-Value < 0.05, **P-Value <0.01, ***P-Value <0.0001

### 3.1 Model 1: Households’ perception of drought

The results of this model showed that households experienced more severe drought conditions in their former places of residence than in their current ones. The impact of past drought conditions was associated with a significant decrease of approximately 17% (*p* = 0.015) in life satisfaction (Table 2). This study also indicates that drought is a primary factor for migration, with 38.42% of households citing it as the main reason for their relocation (Table 1).

### 3.2 Model 2: Occupation of the household head

The findings suggest that farmers and herders experienced significantly lower life satisfaction (β = −0.461, *p* = 0.038) (Table 1). In our study, 33.27% of households were small landowners or agricultural workers who chose to migrate to escape drought conditions. Most of these migrants, due to the lack of specialized skills, were forced to work as construction laborers and day laborers (Table 2).

### 3.3 Models 3 and 4: Rainfall changes and the SPEI index

Quantitative drought indices were considered in these two models, with the results shown in Table 2. We first ran the model comparing rainfall at synoptic meteorological stations with rainfall over the past 30 years (Model 3), which showed that a decrease in rainfall was significantly associated with lower life satisfaction (β = −0.004, *p* = 0.020). However, based on the SPEI index, which considers both temperature and rainfall (Model 4), the decrease was insignificant (β = −0.200). Based on the calculated SPEI index, we classified drought at meteorological stations as mild, moderate, or severe. We found that severe drought significantly reduces life satisfaction by 0.903 (*p* = 0.015) compared to milder droughts.

### 3.4 Economic burden of drought (MRS and WTP)

The marginal rates of substitution (MRS) mentioned in Table 3 indicate how income would change with changes in drought while keeping life satisfaction constant. As we can see, the MRS is higher for households where the head of household was previously employed in agriculture or livestock farming because drought has significantly affected households that relied on agricultural and livestock income. The MRS for households classified as affected by drought based on the Standardized Precipitation-Evapotranspiration Index (SPEI) is very high and noteworthy.

**Table 3:** MRS and WTP based on dollars for different models.

Model	Case Basic		MRS	Willingness to pay based on		
	Drought	Income		GDP per capita (US$)	Household income of the province	Income of the studied samples
Model 1	−0.166	0.780	−0.213	993.544	940.122	734.456
Model 2	−0.461	0.808	−0.570	2663.564	2520.346	1968.983
Model 3	−0.004	1.004	−0.004	18.599	17.599	13.749
Model 4	−0.903	0.922	−0.979	4572.255	4326.408	3379.943

WTP in this study was calculated once based on GDP per capita (in US dollars), once based on household income in Kerman Province. Finally, based on households in the urban slum areas under study in 2022. According to the World Bank report, GDP per capita in 2022 was $4,668.46 [43]; based on the Ministry of Cooperatives, Labor, and Social Welfare’s statistical yearbook, household income was $4,417.44 [44]. The average and standard deviation of the sample’s annual income were $3,451.06 ± $1,177.75.

As shown in Table 4, for a sensitivity analysis, MRS and WTP were calculated for the upper and lower bounds of each drought and income variable. It should be noted that although drought is costly for households, mainly rural households engaged in agriculture or dependent on rainfall, it does not directly imply that individuals must compensate for this cost [21]. As mentioned in part of the discussion, public investment and social structure are important for adapting to drought, especially for small landowners. MRS indicates the extent to which drought reduces income, while WTP shows how much people are willing to spend to offset its impacts. However, given the low and variable income levels of the studied households, the WTP values should be interpreted as indicative measures of welfare loss rather than actual monetary payments that respondents could realistically afford. In this sense, WTP reflects the intensity of drought-related reductions in well-being, not an actionable or policy-level payment expectation.

**Table 4:** Sensitive analysis: The range of changes of drought and income with the upper and lower limits of the derived coefficients.

Model	Coef	Parameters		MRS	Willingness to pay based on		
		Drought	Income		GDP per capita (US$)	Household income of the province	Income of the studied samples
Model 1	β_low_	−0.299	0.222	−1.347	6287.701	5949.615	4648.049
	β_upper_	−0.033	1.338	−0.025	115.141	108.950	85.116
Model 2	β_low_	−0.898	0.250	−3.592	16769.108	15867.444	12396.207
	β_upper_	−0.024	1.366	−0.017	82.023	77.612	60.633
Model 3	β_low_	−0.007	0.373	−0.019	87.612	82.901	64.765
	β_upper_	−0.001	1.636	−0.001	2.853	2.700	2.109
Model 4	β_low_	−1.628	0.265	−6.143	28680.199	27138.084	21201.229
	β_upper_	−0.177	1.58	−0.112	522.986	494.865	386.606

β_low,_ β_upper:_ Coefficient in Low and Upper Level.

## 4. Discussion

### 4.1 Factors influencing life satisfaction

Self-reported life satisfaction is a vital indicator of social well-being and a multidimensional construct [45] that comprehensively evaluates an individual’s life. Unlike the transient emotions associated with immediate experiences, Self-reported life satisfaction is considered relatively stable over time [46–48].

In many studies, determinants of life satisfaction or subjective well-being include age, gender, marital status, education level, income, and perceived government role [49]. However, in our study, the demographic variables influencing life satisfaction with consistent effects across all models include the diminishing impact of aging and, on the other hand, the positive effects of socio-economic status, health, and religion.

A slight but statistically significant adverse effect of age on life satisfaction observed in this study is likely due to increased health issues, reduced income and social networks, and the cumulative impacts of environmental and financial stressors among households residing in urban slum areas. This pattern aligns with observed trends, particularly in developing countries, though it may vary across nations’ socioeconomic conditions, health, and cultural contexts. For instance, in highly developed countries, the relationship between life satisfaction and age often follows a U-shaped curve, with life satisfaction declining during middle age and increasing again in later years [50–52].

The findings highlight the critical role of economic conditions in shaping life satisfaction. As noted, income is the most influential variable, with the most significant impact on life satisfaction (0.94). Similarly, studies have emphasized that income enhances life satisfaction by fulfilling basic needs, enabling access to social services, and improving mental health [53–55]. Income is one of the strongest predictors of subjective well-being across countries, reinforcing the idea that economic resources can shield individuals from stress and hardship [56].

Interestingly, while economic conditions had a significant positive impact, the adverse effect of water stress on life satisfaction was not statistically significant. This suggests that, even though water scarcity poses clear environmental challenges, the need to maintain economic stability may overshadow its effects. Households might prioritize securing sustainable livelihoods and income stability over addressing environmental stressors [57]. A possible explanation for why economic status outweighed the negative impact of water stress in our study is that households may need to allocate financial resources to access high-quality, potable water. On the other hand, water scarcity can impose significant costs on households, including health risks, economic losses, and social conflicts [58,59].

Social capital is also recognized as a key factor and an important predictor of life satisfaction, influencing an individual’s overall sense of well-being and contentment. Perceived social support has been identified as a contributor to increased life satisfaction [60].

The findings indicate that socioeconomic factors play a more prominent role in household life satisfaction than individual-level characteristics, which are overshadowed by macro-level determinants. While economic well-being constitutes a significant determinant of subjective well-being, it is not sufficient in isolation. The effectiveness of economic factors depends on mediating and moderating variables, such as social support, religious beliefs, social security, and environmental security (including water scarcity-induced tensions) [61]. This complex interplay is particularly salient in the context of water stress, where economic pressures stemming from water scarcity can affect household life satisfaction through multiple economic, social, and psychological channels. These findings underscore the need to adopt a multidimensional approach to policies aimed at addressing water stress, mitigating its drought-related impacts, and enhancing household resilience.

### 4.2 Effects of drought on life satisfaction

The effects of drought on life satisfaction (LS) were assessed using two subjective and two objective approaches to ensure the validity of the findings. In the first subjective approach, in which drought was measured by household perceptions, drought significantly affected LS. Other studies have also shown that perceived environmental stress can shape mental health outcomes even more directly than objective metrics [62].

Climate change cannot always be measured through a single method, objective tools, or economic analyses, as individuals may hold conflicting values. Since people perceive the world differently, they prioritize different values. Given the growing impacts of climate change, such as drought, future generations will likely have a more tangible, noticeable, and distinct understanding of the relationship between humans and the environment in the coming decades. Individuals’ values regarding issues like climate change shape their priorities for response or adaptation actions [62].

Due to their dependence on weather patterns for their livelihoods, Farmers’ greater awareness of drought reflects their subjective experiences, including economic insecurity and psychological distress [63]. Farmers’ perception of drought, influenced by their reliance on natural resources, is likely to heighten concerns about income stability and food security—both key components of life satisfaction [64].

Interestingly, the model incorporating rainfall variations showed a small but statistically significant effect on life satisfaction, with a coefficient of −0.004. While the effect size is small, its significance indicates that even minor rainfall fluctuations can affect household mental health, particularly in drought-prone areas [65]. The small effect size may be attributed to households migrating from their original residences, with a minimum of 1 year and a maximum of 10 years.

However, the SPIE classification based on city of origin, categorized into mild, moderate, and severe drought levels, showed a significant adverse effect (−0.90) on life satisfaction, particularly among households from areas severely affected by drought. This finding aligns with research by Berlemann and Steinhardt (2017) on drought-induced migration and its long-term psychological costs [66].

Severe drought disrupts economic stability and weakens the social fabric and cultural continuity of communities, which are often integral to identity and personal well-being [67]. For migrants from severely affected areas, the combined trauma of displacement and livelihood loss may exacerbate feelings of dissatisfaction and lower overall life satisfaction.

In their study, Luong et al. found that an additional year of exposure to drought conditions was associated with a statistically significant decrease in life satisfaction of 0.083 units. However, exposure to drought, as defined by SPEI, did not significantly affect life satisfaction (LS) [68].

The findings reveal that subjective drought measures exert a more substantial influence on life satisfaction than objective indices, consistent with risk perception theory [38]. Farm households that directly experienced drought reported significantly greater reductions in life satisfaction (approximately 2.5 times larger than the effect of risk perception), likely reflecting direct economic losses, forced migration, and psychological distress. This aligns with Lohmann et al., who found that the adverse effect of severe drought damage on life satisfaction was approximately three times greater than the positive effect associated with a doubling of household assets [69].

The weaker effect of precipitation variability may indicate that other climatic factors (temperature, evapotranspiration) and access to alternative water sources mediate households’ experience of water scarcity [70]. Spatial analysis showed that continuous county-level SPEI had no significant effect, but categorical drought severity classification yielded a significant coefficient (−0.9). This pattern suggests threshold effects: gradual differences in drought intensity across Kerman counties do not produce perceptible impacts, but crossing critical thresholds (e.g., moderate to severe drought) generates substantial well-being effects.

The results of this study reveal an interesting relationship between marital status and life satisfaction among households residing in urban slums, with the impact of being widowed or married differing significantly from that of single individuals, depending on the model used. Specifically, household-perception-based and occupational-classification models demonstrate a strong, significant negative relationship between widowhood and life satisfaction. This finding highlights the multidimensional vulnerability of widows, particularly in drought-affected areas. Widowhood often involves profound emotional, social, and economic challenges. The loss of a spouse can lead to social isolation, increased stress, and a diminished support system, all of which can contribute to a decrease in life satisfaction. In rural or agriculturally dependent communities, such as those shown in our study, widowed women may face additional difficulties due to limited access to resources, reduced income potential, and a heavier burden of responsibilities [71–73]. The combined effect of widowhood and drought exacerbates these challenges. For farming households, where drought directly affects livelihood stability, widowed heads or family members may struggle more with financial insecurity and managing daily life.

When assessed subjectively, the consistent finding of a negative impact of widowhood in both drought estimation models indicates that social policies and targeted support measures for this vulnerable population are crucial. Addressing the specific needs of widows, such as strengthening social support networks and providing financial assistance, can help mitigate the impact of widowhood on life satisfaction, particularly in drought-prone areas.

The effects of widowhood and divorce were statistically negligible in models that quantitatively and objectively assessed, such as rainfall changes and the Standardized Precipitation Evapotranspiration Index (SPEI). However, it is noteworthy that married individuals reported higher, statistically significant levels of life satisfaction than single individuals in these models. This finding aligns with previous research showing that married individuals consistently report greater subjective well-being than those who have never married, who in turn report greater well-being than those who were previously married. Marriage is often associated with increased life satisfaction due to emotional, social, and economic support, which may counteract the adverse effects of external stressors such as drought [74,75]. Diener and Seligman (2002) noted that social support is a critical determinant of mental well-being. Married individuals often benefit from greater social and economic stability, which can reduce the stress caused by environmental challenges like drought. This may explain why, in our models, the protective effect of marriage on life satisfaction is significant, while the effect of widowhood has diminished [76].

### 4.3 Willingness to pay (WTP)

The findings of this study provide significant insights into the economic and social responses of households affected by drought, particularly regarding their willingness to pay (WTP) to mitigate its effects. The low WTP observed among respondents aligns with the socio-economic challenges of the sample community, characterized by below-average incomes and reliance on agriculture. This economic outlook forms a “poverty trap” that limits the capacity to invest in drought adaptation measures. This presents a significant challenge: even if households are aware of drought risks and understand them, a lack of financial resources can prevent them from making active investments in adaptation.

Additionally, research by Carroll et al. (2009) found that a spring drought in rural areas led to a notable decrease in life satisfaction, equivalent to an annual income loss of A$18,000. This finding highlights the substantial psychological and future income costs that are often unrecognized [21].

The disparity in Willingness to Pay (WTP) between regions with different socioeconomic statuses emphasizes the need for targeted policy interventions. These should include financial assistance and incentives to bridge the gap between the willingness and ability to invest in drought preparedness. For example, as noted by Aydogdu et al. in Turkey, higher WTP was observed among wealthier households and those with larger landholdings [77]. Our study highlights that, in vulnerable communities such as smallholder communities, support for such initiatives may rely more on external financial aid than on household resources.

The health and economic costs associated with drought also show broader consequences, as observed in studies such as those by De Alwis et al. (2019) and Lohmann and Lechtenfeld (2015), which found that they impose a significant financial burden on households. This aligns with our findings, which show that the pressure of reduced agricultural income leads to reduced spending on essential expenses, including healthcare and nutrition. Addressing these vulnerabilities through comprehensive policy frameworks that enhance economic flexibility and support household stability is crucial [78,79].

As a result, the distinct socio-economic conditions of the study area offer a unique perspective on WTP, which is significantly lower than in more affluent areas. These findings necessitate appropriate policy responses that increase awareness and provide practical support mechanisms. Future research should focus on understanding the long-term impacts of such interventions and exploring innovative budget models to enhance the feasibility of drought adaptation strategies in disadvantaged areas.

Our study has some limitations. One of these is generalizability, as the unique socio-economic and cultural contexts of households in slum areas of Kerman may not reflect those of the broader population. Future studies should focus on inter-regional studies to validate and compare findings. Due to the availability of precipitation and temperature data, we calculated meteorological drought, but could not measure other agricultural drought indices. Lastly, this study does not account for existing or planned policy interventions and adaptation measures that could influence life satisfaction and economic valuations.

One of this study’s strengths is its consideration of water stress in the study areas and the impact of drought over a 30-year horizon on migrant households from the province’s counties who move to the urban slum areas. However, longitudinal studies are recommended to better understand the effects of drought on life satisfaction.

## 5. Policy implications

The findings of this study have direct implications for policymaking at both the micro and macro levels in drought-prone regions.

Enhancing public education and promoting evidence-based policy responses to the growing risk of drought: The findings indicate that households possess a certain level of understanding regarding drought risks. Therefore, at the micro level, policies should focus on promoting participatory climate literacy programs that empower households to adopt sustainable water consumption practices and adaptive strategies. At the macro level, policies should emphasize declaring emergency conditions, assessing preparedness, and collaborating with health and climate experts.

Expand social protection programs: In particular, support vulnerable groups—such as small landowners, agricultural workers, and residents of informal settlements—by enhancing their adaptive capacity, providing targeted financial and technical assistance, and promoting sustainable agricultural practices.

Promote climate adaptation and drought resilience: by investing in decentralized water infrastructure, drought-resistant crops, and community-based water management systems.

Finally, successful drought policy requires interdisciplinary collaboration, robust data systems, and continuous evaluation to adapt to changing climate conditions and evolving socioeconomic vulnerabilities.

## 6. Conclusion

This analytical study examines the economic burden of drought on life satisfaction in slum areas of Kerman. Using the Life Satisfaction Approach, we have highlighted the significant impact of drought on the well-being and economic stability of vulnerable populations. Our findings indicate a substantial economic burden of drought on households, particularly small landowners and agricultural workers, who are forced to abandon their land and jobs and migrate to the slum areas, emphasizing the importance of targeted support for these groups in drought adaptation policies.

Building on these insights, the “Policy Implications” section outlines actionable interventions to enhance drought resilience, mitigate economic hardship, and improve overall life satisfaction. Implementing these evidence-based measures can guide policymakers toward more inclusive and sustainable responses to future drought challenges.
